# Stress responses in flavivirus-infected cells: activation of unfolded protein response and autophagy

**DOI:** 10.3389/fmicb.2014.00266

**Published:** 2014-06-03

**Authors:** Ana-Belén Blázquez, Estela Escribano-Romero, Teresa Merino-Ramos, Juan-Carlos Saiz, Miguel A. Martín-Acebes

**Affiliations:** ^1^Departamento de Biotecnología, Instituto Nacional de Investigación y Tecnología Agraria y AlimentariaMadrid, Spain; ^2^Departamento de Virología y Microbiología, Centro de Biología Molecular “Severo Ochoa”, Consejo Superior de Investigaciones Científicas – Universidad Autónoma de MadridMadrid, Spain

**Keywords:** flavivirus, unfolded protein response, autophagy, dengue virus, West Nile virus, endoplasmic reticulum stress, virus replication

## Abstract

The *Flavivirus* is a genus of RNA viruses that includes multiple long known human, animal, and zoonotic pathogens such as Dengue virus, yellow fever virus, West Nile virus, or Japanese encephalitis virus, as well as other less known viruses that represent potential threats for human and animal health such as Usutu or Zika viruses. Flavivirus replication is based on endoplasmic reticulum-derived structures. Membrane remodeling and accumulation of viral factors induce endoplasmic reticulum stress that results in activation of a cellular signaling response termed unfolded protein response (UPR), which can be modulated by the viruses for their own benefit. Concomitant with the activation of the UPR, an upregulation of the autophagic pathway in cells infected with different flaviviruses has also been described. This review addresses the current knowledge of the relationship between endoplasmic reticulum stress, UPR, and autophagy in flavivirus-infected cells and the growing evidences for an involvement of these cellular pathways in the replication and pathogenesis of these viruses.

## INTRODUCTION

In recent years, the knowledge of virus–host interactions has unveiled multiple connections between virus life cycle steps and a variety of cellular organelles and signaling pathways. Deciphering the complexity of these interactions will provide key information for the control of viral pathogens. This mini-review addresses the current knowledge and challenges for a deep understanding of the interactions of flaviviruses with the endoplasmic reticulum (ER) and two related cellular pathways: the unfolded protein response (UPR) and autophagy.

## FLAVIVIRUS OVERVIEW

The *Flavivirus* genus comprises more than 50 distinct species of enveloped positive single strand RNA viruses. This genus is classified into the *Flaviviridae* family together with *Pestivirus*, *Hepacivirus*, and *Pegivirus* (http://www.ictvonline.org/virusTaxonomy.asp). Flaviviruses include multiple well known human, animal, and zoonotic pathogens such as yellow fever virus (YFV), dengue virus (DENV), tick-borne encephalitis virus (TBEV), Japanese encephalitis virus (JEV), St. Louis encephalitis virus (SLEV), or West Nile virus (WNV), as well as other emerging or re-emerging pathogens such as Usutu virus (USUV) or Zika virus, which are now being considered as potential threats for human and animal health (Weissenbock et al., 2010). As arboviruses (**ar**thropod-**bo**rne **viruses**), most flaviviruses are transmitted by mosquitoes or ticks and maintained in nature through complex infectious cycles that involve different hosts. The variety of symptoms caused by flaviviruses includes jaundice (YFV), febrile illnesses (YFV, DENV, or WNV), hemorrhagic fevers (DENV), or encephalitis (JEV, SLEV, WNV, or TBEV). As a result of different factors, including globalization of travel and trade, climate warming, or changes in land use and vector behavior, different flaviviruses are currently becoming global health threats with DENV being amongst the most prominent human pathogens. In fact, DENV is responsible for up to 50 million infections each year, including 22,000 deaths, mostly among children (http://www.who.int/csr/disease/dengue/impact/en/). There are several vaccines against flaviviruses currently licensed for use in humans (YFV, JEV, TBEV) or animals (WNV, louping ill virus, Wesselsbron virus; Ishikawa et al., 2014). However, there is still a need for specific vaccines or treatments to combat many of these pathogens, i.e., DENV, and a detailed knowledge of flavivirus–host interactions is considered crucial to develop effective therapies.

## ER AND FLAVIVIRUSES: AN INTIMATE RELATIONSHIP

**FIGURE 1:**
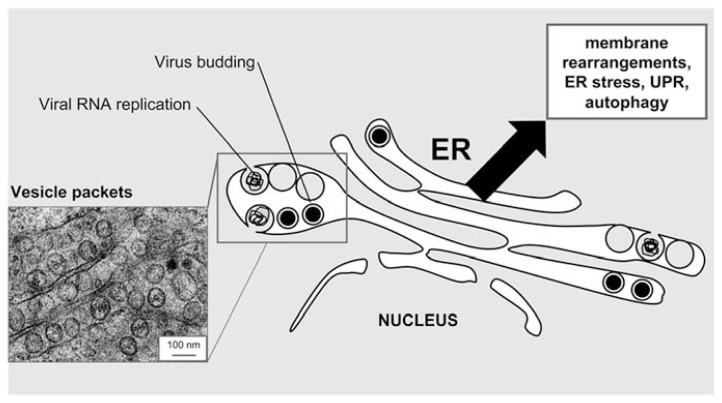
**Schematic view of flavivirus connection with the ER**. The alterations in the ER architecture in flavivirus-infected cells are highlighted. A representative electron micrograph of HeLa cells infected with the flavivirus WNV is shown as an example of these alterations in the ER. Note electron dense virions and vesicle packets. Micrograph courtesy of Miguel A. Martín-Acebes.

Flavivirus replication takes place in association with intracellular membrane structures (**Figure 1**). As other positive-strand RNA viruses, flaviviruses rearrange host cell membranes to build organelle-like structures in order to establish the appropriate environment for viral replication (Paul and Bartenschlager, 2013). The main source of these membranes is provided by the ER where both viral structural and non-structural proteins accumulate (Welsch et al., 2009; Gillespie et al., 2010; Martin-Acebes et al., 2011; Miorin et al., 2013; Junjhon et al., 2014). Membrane reorganizations are driven by viral proteins. These not only induce changes in the protein composition of ER membranes but also in their lipid content (Mackenzie et al., 2007; Heaton et al., 2010; Martin-Acebes et al., 2011; Perera et al., 2012). The formation of the replication complex has been mainly associated with the expression of hydrophobic transmembrane nonstructural proteins NS4A (Roosendaal et al., 2006; Miller et al., 2007) and NS4B (Kaufusi et al., 2014) that are involved in membrane remodeling. The infection induces the formation of membrane vesicles inside the lumen of the ER (an example of WNV-infected cells is depicted in **Figure 1**). These characteristic structures usually referred to as vesicle packets (VPs) or double membrane vesicles (DMVs) have been associated with viral genome replication (Welsch et al., 2009; Gillespie et al., 2010; Miorin et al., 2013; Junjhon et al., 2014). Other flavivirus-induced membrane structures that could also be ER-related are the so-called paracrystalline arrays or convoluted membranes (Mackenzie and Westaway, 2001; Welsch et al., 2009). However, convoluted membranes are not induced in all flavivirus-infected cell types and their specific function in viral infection remains unclear (Junjhon et al., 2014). The newly synthesized viral genomes are enclosed into virions that assemble and bud into the ER, and then traffic through the Golgi complex along the secretory pathway and maturate (Mukhopadhyay et al., 2005) prior to be released from infected cell. In this way, the interaction of flaviviruses with the ER not only provides a replication platform but also the membrane components for the virions (Mukhopadhyay et al., 2005). All these findings make the ER and ER-related pathways key players during flavivirus infection.

## ER, CELLULAR STRESS, AND UPR DURING FLAVIVIRUS INFECTIONS

**FIGURE 2:**
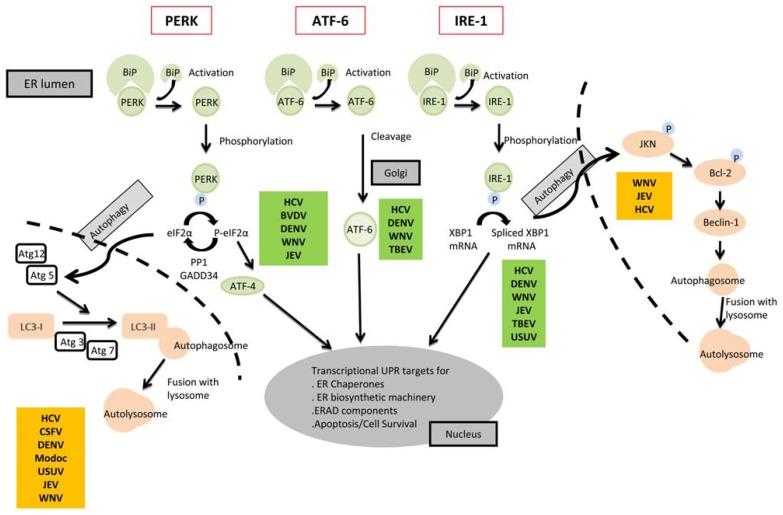
**Cell signaling pathways of the UPR, autophagy connections and flaviviruses**. The three arms of UPR (PERK, ATF-6, IRE-1) are shown in the figure. The viruses from the *Flaviviridae* family whose infection has been related to each process have been noted in the figure. See the text for details.

The ER is an essential organelle involved in many cellular functions including protein folding and secretion, lipid biosynthesis, and calcium homeostasis. A quality control mechanism ensures that only properly folded proteins exit from the ER while incorrectly folded proteins are retained and degraded. The accumulation of misfolded or unfolded proteins can trigger ER stress. To cope with stress, cells activate the intracellular signaling pathway called UPR (Liu et al., 2000). The UPR includes transcriptional induction of genes, attenuation of global protein synthesis, and ER-associated degradation (ERAD). The three main branches of the UPR are the protein kinase-like ER resident kinase (PERK), the activating transcription factor 6 (ATF6), and the inositol-requiring enzyme 1 (IRE1; **Figure 2**; Liu and Kaufman, 2003). These proteins are associated with the ER chaperone BiP/Grp78, which prevents their aggregation and further activation. But the UPR is not only triggered by misfolded proteins, other perturbations can also alter the ER homeostasis such as glucose deprivation, aberrant Ca^2^^+^ regulation or viral infections. Related to the Ca^2^^+^ balance, WNV for example induces a Ca^2^^+^ influx early after infection of cells that has been associated with a virus-induced rearrangement of the ER membrane and activation of different cellular kinases involved in stress response and cell survival, focal adhesion kinase (FAK), mitogen-activated extracellular signal-regulated protein kinase (ERK1/2), and protein-serine kinase B alpha (Akt; Scherbik and Brinton, 2010).

Viruses have evolved to manipulate host UPR signaling pathways to promote viral translation and persistence in infected cells (Chan and Egan, 2005; Tardif et al., 2005; Ke and Chen, 2011; Ambrose and Mackenzie, 2013b; Green et al., 2013). Studies that focused on the *Flaviviridae* family have documented the activation of one or more of the three arms of the UPR. However controversial reports have been published even for the same virus. The reasons for these different results are likely due to differences in the strains or serotypes used, or derived from the use of subgenomic replicons, isolated proteins or complete viruses. For instance, it has been documented that infection by the *Hepacivirus* hepatitis C virus (HCV) leads to the activation of the three UPR signaling pathways (Shinohara et al., 2013) including BiP expression, IRE1 activation, and Xbp-1 splicing (Tardif et al., 2004), ATF6 cleavage (Tardif et al., 2002; Li et al., 2009), eIF2α phosphorylation, and induction of CHOP expression (Chan and Egan, 2005). In contrast, cells harboring a neomycin-adapted subgenomic replicon of HCV that express the nonstructural proteins showed a reduction of eIF2α phosphorylation (Tardif et al., 2002). For the *Pestivirus* bovine viral diarrhea virus (BVDV), the stimulation of proapoptotic effectors with high-level signaling through PERK and eIF2α phosphorylation resulting in CHOP activation and induction of apoptotic effectors caspase 12 and poly ADP ribose polymerase (PARP) has been described (Jordan et al., 2002). Specifically among *Flavivirus*, infection with DENV showed a time dependent activation of the UPR pathways, with PERK activation and eIF2α phosphorylation during early stages of replication that rapidly switched off, with IRE1 and ATF6 upregulation occurring at mid and late stages in the replication cycle, respectively (Pena and Harris, 2011). However, it has also been described the induction of Xbp-1 splicing (Yu et al., 2006; Umareddy et al., 2007; Pena and Harris, 2011), ATF6 cleavage (Umareddy et al., 2007; Pena and Harris, 2011) and activation of GADD34 and CHOP expression leading to apoptosis (Umareddy et al., 2007). In the case of WNV, UPR is activated towards chaperone production and membrane biogenesis to benefit replication (Medigeshi et al., 2007). ATF6 and IRE1 upregulation has also been demonstrated, with Xbp-1s induction, even though the IRE1–Xbp-1 pathway seems to be non-essential for its replication (Medigeshi et al., 2007). In addition to this, WNV strain specific differences regarding regulation of the PERK arm of the UPR have been described. For example, while infection with a WNV attenuated strain prevents PERK-mediated translation and CHOP transcription (Ambrose and Mackenzie, 2010), infection with the highly neurovirulent WNV NY-99 strain upregulates all three pathways of the UPR (Medigeshi et al., 2007) with an early induction of eIF2α phosphorylation and upregulation of downstream apoptotic factors such as CHOP, GADD34, caspase-3, and PARP, which may represent a host defense mechanism to limit viral replication. Other members of the *Flavivirus* genus distinct from DENV and WNV also activate different components of the UPR. For instance, the induction of Xbp-1 splicing after infection with JEV, TBEV, and USUV (Yu et al., 2006, 2013; Blazquez et al., 2013), the expression of CHOP during JEV infection, and the cleavage of ATF6 in TBEV-infected cells (Yu et al., 2006, 2013) have been described.

It is important to highlight the described relevant function of viral proteins of the *Flaviviridae* in the regulation of the UPR. For example, HCV NS4B is a strong regulator of UPR signaling (Zheng et al., 2005; Li et al., 2009), while HCV envelope proteins activate IRE1 and Xbp-1 splicing, and upregulate Bip expression (mainly by E2; Chan and Egan, 2005). WNV NS4A and NS4B strongly induce Xbp-1 transcription and processing when individually expressed, and this ability is directly related to the number of hydrophobic segments they contain (Ambrose and Mackenzie, 2010). In the case of DENV-2, Xbp-1 splicing is induced by NS2B/3 (Yu et al., 2006). Therefore, the role of the UPR during flavivirus infections has been associated with factors contributing to the establishment of an environment more favorable for replication such as chaperone expression, membrane biogenesis, or ATF4-mediated antioxidant and amino acid transporter production. However, some downstream UPR effects such as the inhibition of translation, mRNA decay, production of degradative proteins, or induction of apoptosis are not necessarily beneficial for viral replication (Ambrose and Mackenzie, 2013b). Finally, interaction between the UPR and interferon (IFN) signaling in flaviviral infections has been reported, as ATF6 and IRE1 seem to be required for WNV Kunjin-induced STAT1 phosphorylation and nuclear translocation in response to IFN stimulation (Ambrose and Mackenzie, 2013a). All these findings provide evidence for the multifaceted roles of UPR during flavivirus infections and its connections with cellular metabolism, apoptosis, and innate immunity. These aspects remark the importance of a proper understanding of the interaction of each flavivirus with this cellular signaling pathway.

## STRESS, UPR, AND AUTOPHAGY IN FLAVIVIRUS INFECTED CELLS

Autophagy is a cellular process by which cytoplasmic components are sequestered in double-membrane vesicles and degraded. Autophagy is also intrinsically linked to ER function since the ER provides the membranes involved autophagy (Lamb et al., 2013). There are multiple connections between ER, UPR, and autophagy and changes in ER architecture or composition can trigger autophagy through activation of components of the UPR (Suh et al., 2012; **Figure 2**). By facilitating the removal of damaged organelles and cytoplasmic protein aggregates, autophagy has been proven to be essential for the maintenance of cellular homeostasis (Kudchodkar and Levine, 2009). In addition, this constitutive degradation pathway also plays important roles in development, differentiation, and stress responses (Levine and Klionsky, 2004), and it is an important component of the innate and adaptive immune response elicited against a variety of viral and bacterial pathogens (reviewed in Deretic, 2005; Deretic and Levine, 2009).

The process of autophagy comprises three steps starting with the nucleation and elongation of vesicles to form the phagophore. The edges of phagophore then fuse to assemble the autophagosome. Finally, autophagosomes maturate to autolysosomes by membrane fusion with endosomes (then called amphisomes) or lysosomes (resulting in autolysosomes). Different roles for multiple cellular proteins involved in autophagy have been reported to date. One of the most widely used indicators of upregulation of autophagy is the cytoplasmic aggregation of microtubule-associated protein 1 light chain 3 (LC3), that is modified by its conjugation to phosphatidylethanolamine and targeted to autophagic membranes labeling autophagic vacuoles (Kabeya et al., 2000; Klionsky et al., 2008). An upregulation of the autophagic pathway, characterized by an increase in LC3 modification and its cytoplasmic aggregation, has been noticed following infection by members of the *Flaviviridae* including the flaviviruses DENV, Modoc virus, JEV, USUV (Khakpoor et al., 2009; Panyasrivanit et al., 2009; Heaton and Randall, 2010; Li et al., 2012; McLean et al., 2012; Blazquez et al., 2013; Jin et al., 2013), the hepacivirus HCV (Sir et al., 2008b; Dreux et al., 2009), and the pestivirus classical swine fever virus (CSFV; Pei et al., 2014). Interestingly, upregulation of the autophagic pathway in flavivirus-infected cells can occur without noticeable changes in the levels of the polyubiquitin-binding protein that interacts with LC3 p62/SQSTM1, whose degradation has been described following autophagy induction under certain conditions (Klionsky et al., 2008). This may indicate the unique features of the autophagic response during infections with at least some of these viruses (Beatman et al., 2012; Blazquez et al., 2013). The roles of the autophagic response in flavivirus-infected cells have been associated with varied functions including lipid metabolism reordering to support strong viral replication (Heaton and Randall, 2010), apoptosis inhibition (McLean et al., 2012), innate immunity evasion (Jin et al., 2013), or adequate platforms provision for viral replication during early steps of infection (Khakpoor et al., 2009; Panyasrivanit et al., 2009). Even more, high activation of autophagy has been associated with low neurovirulence of JEV strains (Li et al., 2012), suggesting a protective role of autophagy *in vivo* as already described for other viruses (Orvedahl and Levine, 2008). However, for other flaviviruses like WNV, the induction of an autophagic response in infected cells still remains controversial (Beatman et al., 2012; Vandergaast and Fredericksen, 2012). Nevertheless it seems clear that exogenous stimulation of autophagy via a pro-autophagic peptide can protect against neuronal cell death induced by WNV infection (Shoji-Kawata et al., 2013), thus supporting again a protective role of autophagy *in vivo,* at least against some members of the *Flavivirus* genus.

An induction or manipulation of the UPR has also been described for a wide variety of members of the *Flaviviridae* (**Figure 2**), although relationships between activation of the UPR, membrane remodeling, and autophagy induction have not been addressed in most cases or remain controversial. For instance, the induction of autophagy and UPR has been shown for HCV, but the mechanistic link between the induction of these two cellular processes remains unclear. Some authors have addressed the relationship between both mechanisms, reporting that down-regulation of a variety of UPR modulators inhibits HCV-induced LC3-phosphatidylethanolamine conjugation, a hallmark of autophagic vesicle accumulation (Sir et al., 2008a; Ke and Chen, 2011), or suggesting that HCV-induced eIF2α phosphorylation via PERK activates autophagy (Dreux and Chisari, 2011). Conversely, rapid autophagy induction after HCV infection with stimulation of the UPR at later stages of the infection has been described, implying that autophagy induction is independent of the UPR (Mohl et al., 2012). Supporting the independence of UPR and autophagy, expression of a subgenomic replicon of the pegivirus GB virus B, led to an elevated LC3-II level, but did not induce UPR (Mohl et al., 2012). In the case of flaviviruses, a cause–effect relationship between UPR and autophagy is still lacking. There are contradictory evidences for and against a link between these two processes. For instance, it has been reported that WNV triggers UPR while not always upregulates the autophagic pathway (Vandergaast and Fredericksen, 2012), thus supporting that the induction of the UPR by WNV could be independent of an autophagic response. The only flavivirus protein associated with induction of autophagy has been the DENV NS4A (McLean et al., 2012). This protein is responsible for membrane rearrangements and, in WNV, it is also associated with the induction of the UPR. Although this could support a link between these cellular pathways in flavivirus infection, the involvement of WNV NS4A in autophagy induction has not yet been addressed. All these mixed observations show that there is still a need of new studies to direct evaluate the contribution of UPR to autophagy induction in flavivirus-infected cells.

## CONCLUSION AND FUTURE PERSPECTIVES

The detailed knowledge of the interaction of flaviviruses with the ER is attractive to refine current antiviral strategies against these viruses and to explore novel therapeutic approaches. The view of the ER as a mere replication platform in flavivirus infection should be changed and more emphasis should be given to its profound remodeling of its architecture and composition induced by the infection, including the activation/rearrangement of cellular pathways related to this organelle which are connected with other relevant pathways as apoptosis and innate immunity. In this way, deciphering the puzzle between autophagy, the UPR, and their potential connections could help to build a more complete picture of flavivirus interactions with host cells. An important challenge will be the analysis of autophagy and UPR during flavivirus infection *in vivo* using animal models, of course, having in mind the complex biology of these pathogens that include infection of different host cells within their infectious cycle, which could complicate the interpretation of these studies. In fact, autophagy and UPR currently represent druggable pathways under evaluation for the treatment of multiple human disorders (Suh et al., 2012; Cao and Kaufman, 2013), and recent studies have revealed that pharmacological activation of autophagy can be protective *in vivo* against flavivirus infection (Shoji-Kawata et al., 2013).

## Conflict of Interest Statement

The authors declare that the research was conducted in the absence of any commercial or financial relationships that could be construed as a potential conflict of interest.
